# Nadir CA-125 level as prognosis indicator of high-grade serous ovarian cancer

**DOI:** 10.1186/1757-2215-6-31

**Published:** 2013-04-25

**Authors:** Xia Xu, Yan Wang, Fang Wang, Lizhou Jia, Yiqin Zhou, Fei Deng, Junwei Qu, Bifang Zhou, Aifeng Meng, Bole Fu, Xiaoxiang Chen, Zhiying Qian, Jinhua Wang

**Affiliations:** 1 Department of Chemotherapy, Jiangsu Cancer Hospital, Nanjing, Jiangsu 210009, PR China; 2 Department of Pathology, Jiangsu Province Hospital on Integration of Chinese and Western Medicine, Nanjing, Jiangsu 210028, PR China; 3 Department of Pathology, The University of Texas MD Anderson Cancer Center, Houston, TX 77030, USA; 4 Research Institute of Obstetrics and Gynecology, The third Affiliated Hospital of Guangzhou Medical College, Guangzhou, Guangdong 510150, PR China; 5 Department of Radiotherapy, Jiangsu Cancer Hospital, Nanjing, Jiangsu 210009, PR China; 6 Department of Gynecologic Oncology, Jiangsu Cancer Hospital, Nanjing, Jiangsu 210009, PR China; 7 Department of Nursing, Jiangsu Cancer Hospital, Nanjing, Jiangsu 210009, PR China; 8 State Key Laboratory of Bioelectronics, Southeast University, Nanjing 210096, PR China

**Keywords:** Epithelial ovarian cancer, HG-SOC, CA-125, Prognosis

## Abstract

**Purpose:**

The capacity of nadir CA-125 levels to predict the prognosis of epithelial ovarian cancer remains controversial. This study aimed to explore whether the nadir CA-125 serum levels could predict the durations of overall survival (OS) and progression free survival (PFS) in patients with high-grade serous ovarian cancer (HG-SOC) from the USA and PRC.

**Materials and methods:**

A total of 616 HG-SOC patients from the MD Anderson Cancer Center (MDACC, USA) between 1990 and 2011 were retrospectively analyzed. The results of 262 cases from the Jiangsu Institute of Cancer Research (JICR, PRC) between 1992 and 2011 were used to validate the MDACC data. The CA-125 immunohistochemistry assay was performed on 280 tissue specimens. The Cox proportional hazards model and the log-rank test were used to assess the associations between the clinicopathological characteristics and duration of survival.

**Results:**

The nadir CA-125 level was an independent predictor of OS and PFS (*p* < 0.01 for both) in the MDACC patients. Lower nadir CA-125 levels (≤10 U/mL) were associated with longer OS and PFS (median: 61.2 and 16.8 months with 95% CI: 52.0–72.4 and 14.0–19.6 months, respectively) than their counterparts with shorter OS and PFS (median: 49.2 and 10.5 months with 95% CI: 41.7–56.7 and 6.9–14.1 months, respectively). The nadir CA-125 levels in JICR patients were similarly independent when predicting the OS and PFS (*p* < 0.01 for both). Nadir CA-125 levels less than or equal to 10 U/mL were associated with longer OS and PFS (median: 59.9 and 15.5 months with 95% CI: 49.7–70.1 and 10.6–20.4 months, respectively), as compared with those more than 10 U/mL (median: 42.0 and 9.0 months with 95% CI: 34.4–49.7 and 6.6–11.2 months, respectively). Baseline serum CA-125 levels, but not the CA-125 expression in tissues, were associated with the OS and PFS of HG-SOC patients in the MDACC and JICR groups. However, these values were not independent. Nadir CA-125 levels were not associated with the tumor burden based on second-look surgery (*p* = 0.09). Patients who achieved a pathologic complete response had longer OS and PFS (median: 73.7 and 20.7 months with 95% CI: 63.7–83.7 and 9.5–31.9 months, respectively) than those with residual tumors (median: 34.6 and 10.6 months with 95% CI: 6.9–62.3 and 4.9–16.3 months, respectively).

**Conclusions:**

The nadir CA-125 level was an independent predictor of OS and PFS in HG-SOC patients. Further prospective studies are required to clinically optimize the chances for a complete clinical response of HG-SOC cases with higher CA-125 levels (>10 U/mL) at the end of primary treatment.

## Introduction

Ovarian cancer is the most frequent lethal gynecological cancer in North America and Western Europe; it ranks fifth among the cancers that cause the highest mortality in Chinese women [1-3]. Approximately 22,240 new cases of ovarian cancer in the United States were diagnosed in 2013; 14,030 deaths were caused by the disease in the same year [1]. Despite great progress in the management of ovarian cancer, the mortality rate of ovarian cancer has insignificantly decreased in the past two decades [4]. These data emphasize the need to identify prognostic indicators and more efficient therapeutic strategies for epithelial ovarian cancer (EOC), at least for the high-risk subgroup.

Epithelial ovarian cancer does not usually appear as a single entity but represents a heterogeneous group of distinct disease types, including the serous, endometrioid, mucinous, clear-cell, and undifferentiated carcinomas, as well as in malignant Brenner and mixed mesodermal tumors [5]. We have previously described the different origins, molecular characteristics, and prognosis of high-grade serous ovarian carcinoma (HG-SOC) and all other EOC subtypes [6]. HG-SOC usually represents the archetypical EOC; the high fatality rate of EOC is attributed to HG-SOCs [7].

The CA-125 antigen was developed in the late 1970s and was first reported in 1981 by Bast et al. from mice immunized with the OVCA433 human serous ovarian cancer cell line [8]. This antigen has been evaluated for detecting, monitoring, distinguishing, and observing ovarian or peritoneal malignances [9,10]. Attempts have been made to predict survival using the CA-125 level and also showed that different EOC subtypes have distinct CA-125 levels and prognosis [11,12]. Furthermore, different studies in this research area used distinct recruitment standards. Thus, patients ranged from those with complete clinical response (CCR), as well as persisting and/or progressing disease, at the end of first line treatment to those with a recurrent disease after a PFS that varied from weeks to years. Most of these studies focused on advanced-stage EOC but not on its pathologic type [13-16]. Pathological heterogeneity of EOC was reported to influence the efficiency of the CA-125 level as an indicator in a single-institution study [12]. Type II EOC cases, including HG-SOC, were more conclusive than type I cases when CA-125 was used as a prognosis indicator. Therefore, the evaluation of nadir CA-125 as a prognosis indicator in a large HG-SOC subpopulation is urgently needed.

In the present study, we retrospectively analyzed clinicopathological factors, including CA-125 in patients with HG-SOC who were treated at the MD Anderson Cancer Center (MDACC, USA). We also recruited HG-SOC cases from the Jiangsu Institute of Cancer Research (JICR, PRC) to validate our data. Here, we report the results of this analysis.

## Patients and methods

### Study population

Between January 1, 1990, and February 14, 2011, 616 HG-SOC patients who underwent primary treatment at the MDACC were identified. A total of 80 cases with second-look surgery were available for further study, with detailed exploration information and known CA-125 levels at second-look. Forty-three cases demonstrated a pathological complete response (pCR).

A total of 262 HG-SOC patients from JICR were recruited between January 1, 1992 and December 31, 2011 for validation of the MDACC data. This retrospective study was approved by the Institutional Review Boards of MDACC and JICR. Written informed consent was obtained from the patient for publication of this report and any accompanying images.

### Clinicopathological characteristics

Clinicopathological data was collected using chart review. Data included the age, ethnicity, physical examination, family history, obstetric history, history of present illness, histological type, histological grade, stage, surgical debulking, adjuvant chemotherapy regime, courses of adjuvant chemotherapy, clinical response, second-look operation findings, ascite volume, date of death (if applicable), time of recurrence, and subsequent management [17]. Overall survival (OS) is defined as the time interval from diagnosis until death, or until last follow-up examination of patients who are still alive. Progression free survival (PFS) is the length of time during and after primary treatment wherein the patient’s condition does not worsen. Clinical response and progress were defined according to the Response Evaluation Criteria in Solid Tumors, also known as RECIST, standards [18]. The pathology of all patients was initially reviewed by pathologists from MDACC (J. Liu and J. Zhang) and JICR (X. Y. Xu and N. Hou).

### CA-125 assay

The serum CA-125 concentration was determined using a commercially available Roche immunoassay assay system at the MDACC and JICR clinical laboratories. In clinical practice, a reference value of 35 U/mL is generally considered the upper limit of the normal range.

Tissue samples for the CA-125 immunohistochemical assay were obtained from MDACC (228) and JICR (62) between 1990 and 2001. CA-125 staining was semi-quantitatively assessed in accordance with a previously described standard [19]. The microarray slides of the immunostained tissue were scored using computerized digital analysis (Ariol SL-50; Applied Imaging, California). For the statistical analysis, all cases demonstrating the total integrated optical density (mean ± SE) were grouped together based on a scale of 0 to 3.

We defined the baseline CA-125 level as the level at the start of diagnosis. The nadir CA-125 level includes the observed values during the two-week interval after the first evaluation. The CA-125 level during a relapse includes those observed in the two-week interval after the relapse. The relationship between the nadir CA-125 level and the duration of survival was explored: (1) as a continuous variable in the Cox regression analysis and (2) as a dichotomous variable around the median in the log-rank test.

### Statistical analysis

The Cox proportional hazards model was used to assess the association between survival and the absolute serum CA-125 level as well as the decreasing kinetics. Step-wise regression techniques were used to build multivariate models, with a cut-off of 0.15 to remain in the model. Associations were regarded as significant if the two sides had *p* < 0.05. The Kaplan–Meier estimates were calculated and compared using the log-rank test for OS and PFS values stratified by various prognostic factor categories. All analyses were conducted using SAS (version 9.0; SAS Institute, Carey, North Carolina, USA).

## Results

### Patient characteristics

A total of 616 HG-SOCs from MDACC and 262 cases from JICR were recruited in this study. The clinicopathological characteristics of the patients are described in Table 1. The median follow-up of the survivors was 38 months (interquartile range, 20.2 months to 73.0 months) for the patients from MDACC. The median follow up of the survivors was 35 months (interquartile range, 18.4 months to 66.2 months) for the patients from JICR.

**Table 1:** Patient characteristics of the study population

**Characteristic**	**Percentage (%)/median (range)**	
	**MDACC (*n*** **= 616)**	**JICR (*n*** **= 262)**
**Age (years)**	60.3 (20–92)	62.1 (22–85)
**Baseline CA-125 level (U/mL)**	800 (7–33423)	927 (5–24880)
**Nadir CA-125 level (U/mL)**	10 (4–35)	10 (2–35)
**Ethnic group**		
White	481(78.1)	0 (0.0)
Black	35 (5.7)	0 (0.0)
Hispanic	76 (12.3)	0 (0.0)
Eastern Asian	16 (2.6)	262 (100.0)
Others *	8 (1.3)	0 (0.0)
**Surgical residual**		
<1 cm	366 (59.4)	142 (54.2)
1–2 cm	19 (3.1)	7 (2.7)
>2 cm	154 (25.0)	76 (29.0)
Unknown	77 (12.5)	37 (14.1)
**FIGO stage**		
I	49 (8.0)	21 (8.0)
II	36 (5.8)	15 (5.7)
III	410 (66.6)	171 (65.3)
IV	125 (20.3)	52 (19.8)
Unknown	4 (0.6)	2 (0.8)
**Neo-adjuvant chemotherapy**	133 (21.2)	186 (71.0)

FIGO the International Federation of Gynecology and Obstetrics.

Others* including 5 Middle Eastern, and 3 Indian cases.

### Nadir CA-125 level was a prognosis indicator in HG-SOCs

The median baseline CA-125 level was 800 U/mL (interquartile range: 322 U/mL to 2871 U/mL) and the median nadir CA-125 level was 10 U/mL (range: 4 U/mL to 35 U/mL) in MDACC HG-SOCs. The median baseline and nadir CA-125 levels in JICR cases were 927 U/mL (interquartile range: 428 U/mL to 3266 U/mL) and 10 U/mL (range: 2 U/mL to 35 U/mL), respectively. The rate of positive CA-125 expression in HG-SOC tissues was 91.6%.

Among all the related clinic characteristics that were included in the univariate analysis of MDACC patients, the nadir and baseline CA-125 level was a predictor of PFS and OS (*p* < 0.01 for all values). Likewise, the decreasing CA-125 kinetics during primary treatment and neoadjuvant chemotherapy predicted the PFS (*p* = 0.01 and *p* = 0.02, respectively) but not the OS (p = 0.22 and p = 0.10, respectively), as described in Table 2. The nadir CA-125 level was an independent predictor of PFS and OS (*p* < 0.01 for both). However, the same does not apply to the baseline CA-125 level, decreasing kinetics, and neoadjuvant chemotherapy, as described in Table 3.

**Table 2:** Univariate analysis of survival-related characteristics in HG-SOCs

**Variable**	**PFS (OR, 95% CI)**		**OS (OR, 95% CI)**	
	**MDACC**	**JICR**	**MDACC**	**JICR**
**FIGO stage**				
I	1.00 (reference)	1.00 (reference)	1.00 (reference)	1.00 (reference)
II	1.11 (0.44–2.81)	1.27 (0.63–3.95)	1.31 (0.45–3.77)	1.62 (0.65–3.99)
III	2.66 (1.25–5.66)	4.19 (1.97–9.64)	3.65 (1.56–9.17)	4.79 (2.26–10.14)
IV	3.03 (1.37–6.70)	6.57 (3.05–13.69)	5.38 (2.20–13.20)	7.03 (3.28–15.05)
**Ascites**				
No	1.00 (reference)	1.00 (reference)	1.00 (reference)	1.00 (reference)
Yes	1.73 (1.22–2.47)	1.93 (1.52–2.42)	1.92 (1.42–2.60)	2.04 (1.61–2.58)
**Residual tumors**				
No	1.00 (reference)	1.00 (reference)	1.00 (reference)	1.00 (reference)
Yes	1.54 (1.10–2.14)	1.68 (1.15–2.40)	1.99 (1.63–2.63)	2.10 (1.67–2.56)
**Neo-chemotherapy**				
Yes	1.00 (reference)	1.00 (reference)	1.00 (reference)	1.00 (reference)
No	1.38 (1.01–1.95)	1.45 (1.14–1.78)	1.33 (0.40–3.56)	1.12 (0.84–1.60)
**Chemotherapy, including paclitaxel**				
Yes	1.00 (reference)	1.00 (reference)	1.00 (reference)	1.00 (reference)
No	1.01 (0.82–1.47)	1.12 (1.00–1.44)	1.30 (0.41–4.10)	1.18 (0.80–1.63)
**CA-125, decreasing kinetics**				
≥1/32	1.00 (reference)	1.00 (reference)	1.00 (reference)	1.00 (reference)
<1/32	1.12 (1.06–1.47)	1.50 (1.23–1.85)	1.17 (0.62–2.27)	1.09 (0.67–2.21)
**Baseline CA-125**	1.02 (1.01–1.06)	1.03 (1.01–1.06)	1.03 (1.01–1.05)	1.06 (1.02–1.10)
**CA-125 level at relapse**	1.01 (1.00–1.01)	1.01 (1.00–1.02)	1.01 (1.00–1.01)	1.01 (1.00–1.13)
**Nadir CA-125**	1.04 (1.02–1.08)	1.06 (1.02–1.09)	1.02 (1.01–1.04)	1.04 (1.01–1.07)

**Table 3:** Multivariate analysis of survival-related characteristics in HG-SOCs

**Variable**	**PFS (OR, 95% CI)**		**OS (OR, 95% CI)**	
	**MDACC**	**JICR**	**MDACC**	**JICR**
**FIGO stage**				
I	1.00 (reference)	1.00 (reference)	1.00 (reference)	1.00 (reference)
II	1.07 (0.35–3.14)	1.22 (0.54–4.19)	1.24 (0.40–3.98)	1.53 (0.63–4.24)
III	1.83 (1.18–4.29)	3.24 (1.61–8.93)	2.28 (1.14–8.65)	3.56 (2.02–8.27)
IV	2.26 (1.24–5.25)	4.37 (2.52–11.05)	3.77 (2.08–10.24)	5.13 (2.72–12.27)
**Ascites**				
No	1.00 (reference)	1.00 (reference)	1.00 (reference)	1.00 (reference)
Yes	1.40 (1.16–2.03)	1.85 (1.47–2.73)	1.73 (1.26–2.49)	2.01 (1.55–2.47)
**Residual tumors**				
No	1.00 (reference)	1.00 (reference)	1.00 (reference)	1.00 (reference)
Yes	8.54 (4.24–16.82)	9.12 (4.50–17.87)	6.24 (3.19–16.39)	7.35 (3.72–14.08)
**Neo-chemotherapy**				
Yes	1.00 (reference)	1.00 (reference)	1.00 (reference)	1.00 (reference)
No	1.28 (0.88–2.24)	1.27 (1.19–1.42)	1.26 (0.36–6.22)	1.17 (0.75–1.93)
**Chemotherapy, including paclitaxel**				
Yes	1.00 (reference)	1.00 (reference)	1.00 (reference)	1.00 (reference)
No	1.00 (0.61–2.62)	1.07 (0.86–1.40)	1.21 (0.38–4.77)	1.13(0.74–1.86)
**CA-125, decreasing kinetics**				
≥1/32	1.00 (reference)	1.00 (reference)	1.00 (reference)	1.00 (reference)
<1/32	1.06 (0.84–1.83)	1.37 (1.14–1.92)	1.10 (0.56–2.84)	1.02 (0.65–2.85)
**Baseline CA-125**	1.00 (0.98–1.02)	1.00 (0.98–1.02)	1.00 (0.97–1.03)	1.00 (0.97–1.03)
**CA-125 level at relapse**	1.00 (0.97–1.04)	1.00 (0.98–1.01)	1.01 (0.98–1.02)	1.01 (0.99–1.02)
**Nadir CA-125**	1.02 (1.00–1.04)	1.02 (1.00–1.03)	1.03 (1.01–1.06)	1.03 (1.00–1.05)

We further explored the value of the nadir CA-125 level for predicting OS and PFS durations in HG-SOCs from JICR. The nadir CA-125 level remained an independent predictor of PFS and OS (*p* < 0.01 for both). However, the same cannot be said for the baseline CA-125 level, decreasing kinetics, and neoadjuvant chemotherapy, as described in Tables 2 and 3.

### Nadir CA-125 level can predict prognosis of EOC

**Figure 1:**
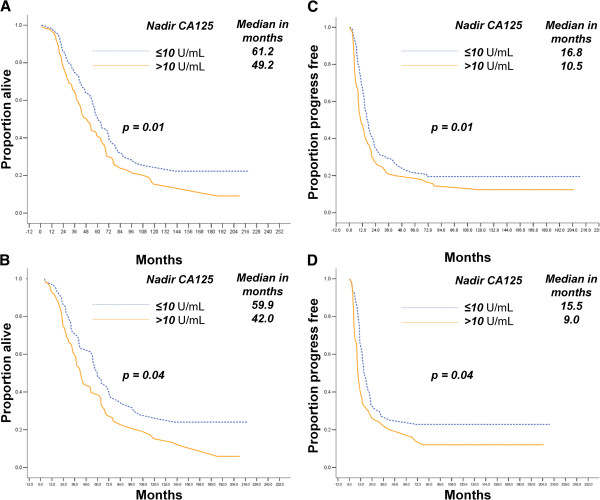
Nadir CA-125 level is associated with OS (A, B) and PFS (C, D) in HG-SOCs from the MDACC (A, C) and JICR (B, D) groups.

Patients with a nadir CA-125 level lower than or equal to the median level (10 U/mL) had longer OS and PFS than those with levels higher than 10 U/mL in MDACC patients (*p* = 0.01 for both), as shown in Figure 1A and 1C, respectively. Meanwhile, Figure 1B and 1D show that a lower nadir CA-125 level in the JICR cases was associated with longer OS and PFS of HG-SOC (*p* = 0.04 for both).

**Figure 2:**
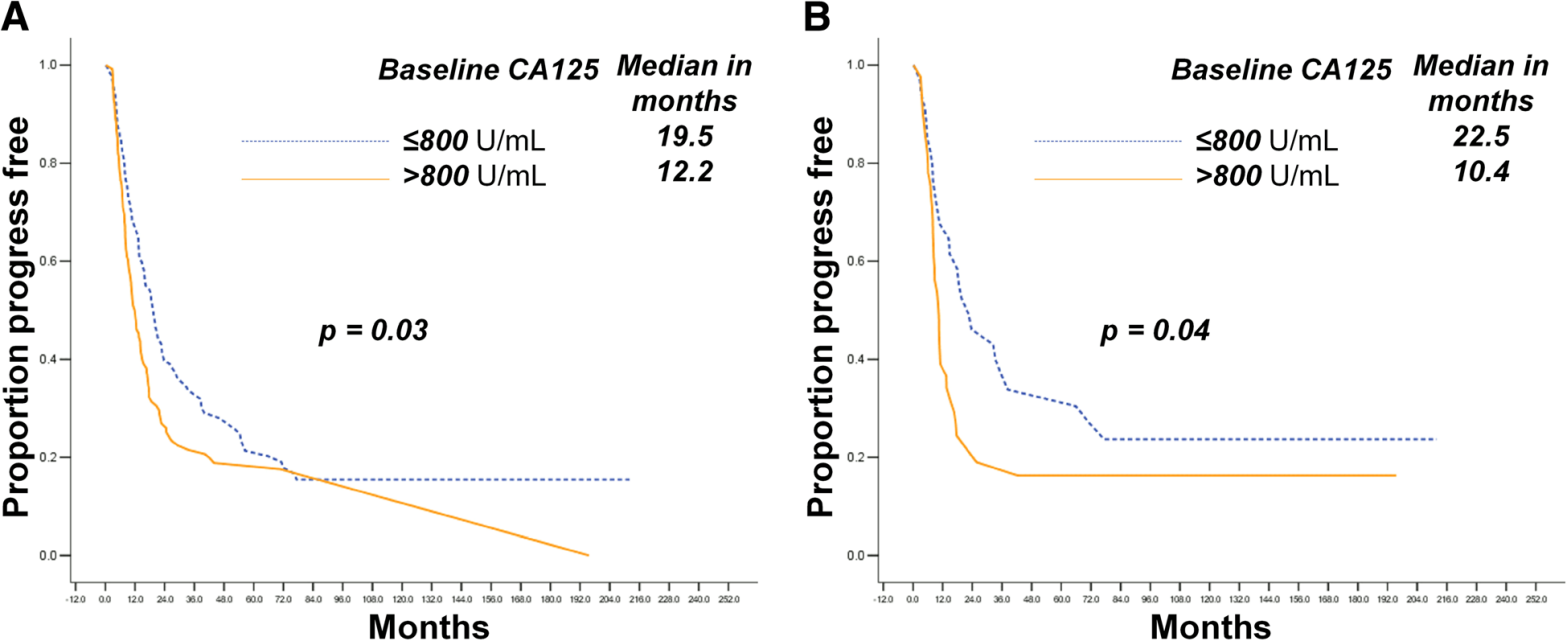
Baseline CA-125 level is associated with PFS in HG-SOCs from the MDACC (A) and JICR (B) groups.

We found a baseline CA-125 level lower than 800 U/mL was associated with longer PFS in the MDACC and JICR cases (*p* = 0.03 and *p* = 0.04, respectively). However, the same does not apply to the OS durations, as shown in Figure 2. Notably, the nadir CA-125 level (*p* < 0.01), but not the CA-125 level at relapse (*p* = 0.12), was an independent OS indicator in the ovarian group from MDACC, as revealed by the multivariate regression analysis in Table 3.

### Nadir CA-125 cannot predict residual tumors in HG-SOCs

**Figure 3:**
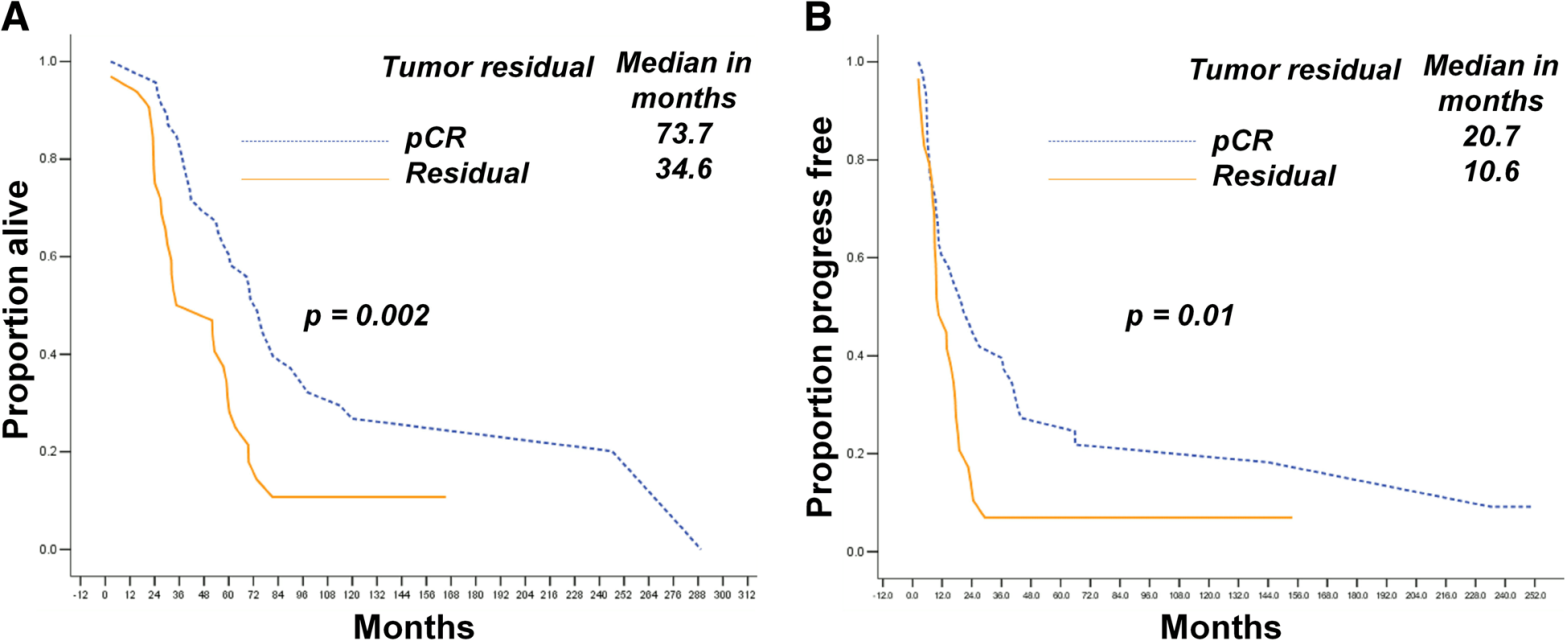
Patients who underwent pCR have longer OS (A) and PFS (B) than other HG-SOCs from the MDACC group.

Among the CCR HG-SOCs from MDACC that underwent a second-look, the pCR cases had longer OS (*p* < 0.01) and PFS (*p* = 0.01) than those with residual lesions, as shown in Figure 3.

To evaluate the potential correlation between the nadir CA-125 level and the residual tumors in second-look surgery, we performed binary logistic regression analysis among these HG-SOC cases. No significant correlations were found (*p* = 0.09; Table 4).

**Table 4:** Logistic regression of residual tumor-associated factors in HG-SOCs

**Factors**	**Univariate**		**Multivariate**	
	**Exp(β)**	**Sig**	**Exp(β)**	**Sig**
Age	1.01	0.15	1.30	0.42
Ascites	1.47	0.02	2.22	0.24
Stage	2.52	0.00	1.96	0.04
Nadir CA-125	1.05	0.01	1.02	0.09

## Discussion

The use of higher nadir CA-125 levels below the upper normal limit to indicate poorer prognosis in CCR EOC patients and the consequent need for further consolidation or maintenance therapy has been a long-standing issue [20]. The primary therapy of EOC patients commonly ends after standard treatment, most including cytoreduction surgery and adjuvant chemotherapy. Several studies have suggested that the CA-125 level may be used to stratify high-risk recurrent patients [13]. As heterogeneous groups, different EOC pathological types present distinct clinical characteristics that indicate patient prognosis, including the CA-125 level. The use of a uniform reference value or interval may not be appropriate for estimating the prognosis of all types of EOCs. The inconsistent results from different studies may be also partially attributed to the unspecific use of CA-125 for all EOC types.

In the present study, we found that the nadir CA-125 level is an independent prognosis indicator of HG-SOC. The OS and PFS varied between the subgroups with median nadir serum CA-125 levels (10 U/mL). Although the CA-125 level has been associated with EOC prognosis, no consensus has been reached regarding a fixed cut-off value for the nadir CA-125 level when predicting prognosis. Several studies have suggested a CA-125 level based on the observed median value [21]. Others used an arbitrary cut-off value, such as 10 U/mL and 12 U/mL, or an interval for the stratification of cohorts, such as 5 U/mL, 21 U/mL, and 18 U/mL [12,16,22-24]. However, the median and arbitrary cut-off values vary among the different EOC subtypes. The median nadir CA-125 level of HG-SOC was 10 U/mL in both MDACC and JICR populations. Furthermore, the OS and PFS were longer for the pCR subgroup than for patients with residual tumors confirmed by second-look surgery; however, this issue remains controversial [25-30]. Although the serum CA-125 level is known to reflect the tumor burden, our results did not confirm its role in determining the minimal degree of residual disease [31-33]. Given that only 80 of 616 patients (13.0%) were included in the analysis, the validity of our findings for the population with HG-SOC is unclear.

Based on the consideration that CA-125 can predict prognosis, the conventional concept of the CCR as the end of primary treatment needs further evaluation [12]. Identifying the best therapy and defining the patient groups that might best benefit from a consolidation or maintenance strategy are equally challenging, given that the present strategy assumes that all HG-SOC cases will recur. Thus, clinicians will be able to advise patients who achieve CCR after primary chemotherapy by determining the therapeutic ratio of possible harm versus potential benefit for an individual patient. The same recruitment standards and analysis methods were used to study HG-SOC from two different cancer centers. Thus, the bias in this study was reduced to a certain extent. The nadir CA-125 level associated with prognosis does not reflect the presence of residual tumors. CA-125 is ineffective for minor tumor burdens.

The baseline CA-125 is found a significant but not independent variables. Actually, higher decline rate of CA-125 means higher baseline level in CCR ovarian cancer with negative CA-125 level (<35 U/mL). CA-125 level >10 U/mL meanwhile decreasing more than 97.6% decreasing kinetics <1/32) indicates poorer OS and PFS, in another words, baseline CA-125 level more than or equal to 424-1484 U/ml indicate more relapse and death. Our results suggest that for patients even achieved CCR, we need pay more attention to those with high initial CA-125 level.

This retrospective analysis has several limitations. First, given the long survival durations of our study population from the two institutions, the heterogeneity of treatment strategies used throughout the 28-year study period, the emergence of new treatment regimes, such as paclitaxel-based chemotherapy and molecular target therapy [34,35], the influence of the therapy and evaluate strategy is difficult to exclude. Second, we focused on the nadir CA-125 level in HG-SOCs. However, 8.4% negative tissue expression and 11.2% normal serum level were observed for CA-125.

Our study supports the emerging evidence showing that the nadir CA-125 level in HG-SOCs may be an independent prognostic factor for both OS and PFS. The application of CA-125 strata for future prospective trials of HG-SOC consolidation or maintenance should be considered.

## Abbreviations

CCR: Complete clinical response; OS: Overall survival; PFS: Progression free survival; EOC: Epithelial ovarian cancer; pCR: Pathologic complete response.

## Competing interests

The authors declare that they have no competing interests.

## Authors’ contributions

XX, XXC, ZYQ and JHW participated in drafting the full manuscript and writing of this manuscript. YW, FW and LZJ partly participated in clinical study design, coordination and data analysis. YQZ, FD, BFZ, AFM and BLF participated in collecting data, creating figures and tables. XX, FW and FW contributed by writing specific sections of this manuscript. ZYQ and JHW provided advice and participated in revising the manuscript. XXC participated in substantial contribution to conception and revising it critically for important intellectual content. All the authors in this manuscript have read and approved the final version.
